# Autophagy Activated by Bluetongue Virus Infection Plays a Positive Role in Its Replication

**DOI:** 10.3390/v7082838

**Published:** 2015-08-17

**Authors:** Shuang Lv, Qingyuan Xu, Encheng Sun, Tao Yang, Junping Li, Yufei Feng, Qin Zhang, Haixiu Wang, Jikai Zhang, Donglai Wu

**Affiliations:** State Key Laboratory of Veterinary Biotechnology, Harbin Veterinary Research Institute, Chinese Academy of Agricultural Sciences, Harbin 150001, China; E-Mails: shuanglv8728@163.com (S.L.); x_qingyuan@163.com (Q.X.); ecsunhvri@163.com (E.S.); yangtao04@126.com (T.Y.); lijunping916@163.com (J.L.); fyf_anna@163.com (Y.F.); zq_dolphin11@163.com (Q.Z.); wanghaixiu217@163.com (H.W.); 15776628009@163.com (J.Z.)

**Keywords:** bluetongue virus (BTV), autophagy, interplay, replication

## Abstract

Bluetongue virus (BTV) is an important pathogen of wild and domestic ruminants. Despite extensive study in recent decades, the interplay between BTV and host cells is not clearly understood. Autophagy as a cellular adaptive response plays a part in many viral infections. In our study, we found that BTV1 infection triggers the complete autophagic process in host cells, as demonstrated by the appearance of obvious double-membrane autophagosome-like vesicles, GFP-LC3 dots accumulation, the conversion of LC3-I to LC3-II and increased levels of autophagic flux in BSR cells (baby hamster kidney cell clones) and primary lamb lingual epithelial cells upon BTV1 infection. Moreover, the results of a UV-inactivated BTV1 infection assay suggested that the induction of autophagy was dependent on BTV1 replication. Therefore, we investigated the role of autophagy in BTV1 replication. The inhibition of autophagy by pharmacological inhibitors (3-MA, CQ) and RNA interference (siBeclin1) significantly decreased viral protein synthesis and virus yields. In contrast, treating BSR cells with rapamycin, an inducer of autophagy, promoted viral protein expression and the production of infectious BTV1. These findings lead us to conclude that autophagy is activated by BTV1 and contributes to its replication, and provide novel insights into BTV-host interactions.

## 1. Introduction

Bluetongue is an insect-transmitted disease that infects wild and domestic ruminants, especially cattle and sheep [1]. The causative agent, bluetongue virus (BTV), a member of the *Orbivirus* genus in the *Reoviridae* family, is an architecturally complex arbovirus. The virion is a non-enveloped icosahedral particle consisting of the outermost double-capsids and ten genomic double-stranded RNA (dsRNA) segments that encode seven structural proteins (VP1 to VP7) and four nonstructural proteins (NS1 to NS4) [2,3]. Due to the impact of bluetongue on international trade and economic development, BTV has been the subject of extensive molecular virology and structural biology studies [4,5,6,7]. Even so, the underlying mechanisms guiding the interactions between the virus and host remain poorly understood.

Viruses exploit multiple mechanisms to modulate host cells, presumably for replicative advantages. Among them, autophagy is a highly conserved catabolic process that mediates the clearance of long-lived proteins and damaged organelles via a lysosomal degradative pathway, encompassing macroautophagy, microautophagy and chaperone-mediated autophagy [8,9]. Many autophagy-related genes (ATG) are involved in this intracellular degradation process to maintain the homeostasis of cells. These genes work in coordination to regulate autophagy, including the formation of autophagosomes and their fusion with lysosomes [10]. Under normal conditions, autophagy proceeds at a basal level, but it is markedly activated in response to a variety of extracellular and intracellular stimuli, including nutrient starvation, energy depletion, reactive oxygen stress and microbial infection [11,12,13].

Many recent studies have reported that viral infections are involved in the complex autophagic process. Although autophagy commonly serves as a defense mechanism against viral infection [14,15], some viruses appear to be unaffected by autophagy, and many viruses have evolved to escape or to exploit this mechanism to promote their survival and replication in different ways [8,16,17,18]. Thus, the role of autophagy in host-virus interactions is diverse for different viruses.

Regarding BTV, we speculate that autophagy is likely to be involved in BTV infection based on several experimental clues: (i) In one study, activated host autophagy appeared to inhibit post-BTV infection when cells were treated with two compound agents, C003 and C052 [19], but a more specialized and systematic verification has not been reported. In addition, another member of the *Orbivirus* genus, epizootic hemorrhagic disease virus (EHDV), can induce autophagy in cultured mammalian cells [20]; (ii) Recent research findings have described the modulation of autophagy by mammalian reovirus (MRV) and by avian reovirus (ARV), which both belong to the *Reoviridae* family and share a similar virus structure [13,21,22]; (iii) Finally, crosstalk has been observed between apoptosis and autophagy pathways [23,24]. Exploitation of the host apoptosis pathway is a critical strategy utilized by BTV for its pathological effects, as evidenced by a recent experimental system that demonstrated that BTV infection triggers apoptosis in mammalian cells and that the uncoating of BTV was required for this process [25,26]. However, to date, the role of autophagy during BTV infection and replication is unknown.

In this report, we provide evidence that autophagy is triggered in permissive BSR cells upon infection by BTV1. Notably, a similar phenomenon was observed in primary natural host cells. By regulating autophagy via pharmacological treatment and RNA interference, we demonstrate that autophagy plays a positive and critical role in BTV replication. A better comprehension of the interactions between BTV and host autophagic responses will provide new insights into viral pathogenesis and antiviral drug development.

## 2. Materials and Methods

### 2.1. Cells, Viruses and Plasmids

BSR cells were maintained in Dulbecco’s modified Eagle’s medium (DMEM) supplemented with 5% fetal bovine serum (FBS) and antibiotics (0.1 mg·mL^−1^ of streptomycin and 100 IU·mL^−1^ of penicillin). Primary lamb lingual epithelial cells were maintained in DMEM supplemented with 10% FBS and 1% antibiotics. BTV1 strain SZ97/1 (GenBank No. JN848767) was propagated in BHK-21 cells.

To construct pEGFP-LC3B, the LC3B gene was amplified from BSR cells with primers (LC3F: GTGAATTCTCCGTCCGAGAAGACCTTCAAG; LC3R: AAGTCGACTTACACAGCCATTGCTGTCCCGA) that were designed based on the sequence of LC3B (GenBank No. NM_026160.4) and then cloned into pEGFP-C1 to express LC3B fused with the EGFP protein at its *N*-terminus.

### 2.2. Virus Infection and Titration Assays

BSR cells in 6-well plates were infected with BTV1 at a multiplicity of infection (MOI) of 1 (100 μL per well) for autophagy induction, and the supernatant was removed after absorption for 1 h. The cell monolayers were rinsed three times with sterile phosphate buffered saline (PBS, pH 7.2) and incubated in fresh complete medium at 37 °C for the indicated times. Then, the extracellular viruses from cell supernatants and intracellular viruses from cells treated with multiple freeze-thaws were harvested and serially diluted 10-fold to infect confluent BSR cells in 96-well plates. Finally, cytopathic effects (CPEs) were observed and recorded at 72 h post-infection (hpi). Virus titers were calculated as TCID_50_/mL using the Reed-Muench method.

### 2.3. SDS-PAGE and Western Blot

Following the indicated treatment or transfection, all samples were lysed using Western and IP lysis buffer (P0013, Beyotime, Beijing, China) containing PMSF and Complete Protease Inhibitor Cocktail (04693116001, Roche, Penzberg, Germany). The clarified lysates pelleted by centrifugation were boiled in 5× SDS-PAGE loading buffer for 10 min, and then were subjected to 12% SDS-PAGE gel and then electrotransferred onto NC membranes. The membranes were blocked for 2 h, and incubated with the following primary antibodies for 2 h: Rabbit anti-LC3B antibody (L7543, Sigma, St Louis, MO, USA), rabbit anti-p62/SQSTM1 antibody (P0067, Sigma), mouse anti-actin antibody (TA-09, Zhongshan Golden Bridge, Beijing, China), rabbit anti-Beclin1 antibody (3738S, CST, Danvers, MA, USA) and mouse monoclonal antibodies against the VP2 NS3 and NS1 proteins of BTV1. The membranes were then incubated with IRDye^®^ 800 CW goat anti-mouse IgG or goat anti-rabbit IgG (LiCor BioSciences, Lincoln, NA, USA) as secondary antibodies. The blots were visualized using an Odyssey Infrared Imaging System (LiCor BioSciences).

### 2.4. Transmission Electron Microscopy

Transmission electron microscopy (TEM) was used to observe autophagosomes as described previously [27]. Specifically, BSR cells were collected at 12 and 24 hpi with BTV1 at an MOI of 1. The mock group and rapamycin-treated group were from 24 h samples. Then, the samples were prepared for TEM observation. Autophagosomes were judged as double-membrane vesicles with diameter of 0.5 to 2.0 μm.

### 2.5. Confocal Fluorescence Microscopy

For the detection of autophagosomes, BSR cells were grown to 70% confluence in 35-mm glass-bottomed culture dishes (NEST) and then transfected with 2 μg of pEGFP-LC3 using Lipofectamine 2000 (11668-27, Invitrogen, Carlsbad, CA, USA). The cells were disposed with rapamycin or virus as described above at 12 h post-transfection, and then were fixed with cold absolute ethyl alcohol at 24 h post-treatment. The formation of EGFP-LC3 fluorescence dots was observed using a Leica SP2 confocal system (Leica Microsystems, Wetzlar, Germany). The BTV infection was marked using antibody against the BTV NS1 protein, followed by the corresponding secondary antibody conjugated to TRITC. The cell nucleus was counterstained with DAPI.

To observe the effect of chloroquine (CQ) on LC3 puncta, BSR cells transfected by pEGFP-LC3 were infected with BTV in presence or absence of CQ for 24 h at 12 h post-transfection. The cell nucleus was counterstained with DAPI.

To detect autophagic flux, BSR cells were transfected with pEGFP-LC3, infected with BTV or left untreated as described above. After fixment and permeabilization, the cells were stained with 50 nM LysoTracker Red DND-99 (acidic compartments marker) (C1046, Beyotime) for 30 min. After the cells were washed three times with PBS, they were processed to analyze using a confocal microscope.

### 2.6. Preparation of Ultraviolet (UV)-Inactivated BTV1

To obtain a replication-incompetent BTV1 strain, UV inactivation was performed as described previously [28]. Virus preparations (2 mL) were exposed to 254 nm UV, 10 cm from the source, in a 35-mm diameter dish for 40 min. The loss of infectivity of UV-inactivated BTV1 was experimentally verified in BSR cells.

### 2.7. Cell Viability and Drug Treatment Assay

Three drugs, 3-methyladenine (3-MA) (M9281, Sigma), chloroquine (CQ) (C6628, Sigma) and rapamycin (Rapa) (S1842, Beyotime), were used in this experiment to explore the effects of autophagy on the replication of BTV. The cytotoxicity of these drugs at optimal concentrations (10 mM 3-MA; 20 μM CQ; 100 nM Rapa) was tested using a WST-1 cell proliferation and cytotoxicity assay kit (C0035, Beyotime) according to the manufacturer’s protocol. Next, BSR cells were pretreated with optimal concentrations of Rapa, 3-MA for 2-4 h and were not pretreated with CQ prior to BTV infection. Subsequently, the cells were infected with BTV1 as above and further incubated in fresh media in the absence or presence of these drugs at the same concentrations as for the pretreatments. Corresponding DMSO or ddH2O were used as vehicle controls. At the indicated times, the virus from the supernatants and cells were collected and the viral protein expression were detected as above.

### 2.8. RNA Interference of Beclin1

To further determine the role of autophagy induced by BTV during its life cycle, small interfering RNAs (siRNA) were synthesized by Shanghai GenePharma Co., Ltd. (Shanghai, China) to target Beclin1. The siRNA sequences for targeting Beclin1 (siBeclin1) were as follows: GAUGGUGUCUCUCGAAGAUTT (sense) and AUCUUCGAGAGACACCAUCTT (antisense). To evaluate the efficiency of the siRNAs, 80 pmol siBeclin1 and negative control siRNA (siNC) were transfected into BSR cells cultured in a 12-well plate using Lipofectamine 2000 following the manufacturer’s instructions. At 24 h post-transfection, the knockdown efficiency was determined by examining endogenous Beclin1 expression by Western blot. The cells were infected with BTV1 (MOI = 1, 50 μL per well) at 24 h post-transfection and incubated for 24 h before samples were collected to determine the virus titer and viral protein expression.

### 2.9. Statistical Analysis

Statistical analyses were performed using GraphPad Prism 6.0 software. Student’s *t*-test was used to analysis significance. *p* values of <0.05 were considered significantly different.

## 3. Results

### 3.1. Notable Autophagy is Triggered by BTV1 Infection in BSR Cells

**Figure 1:**
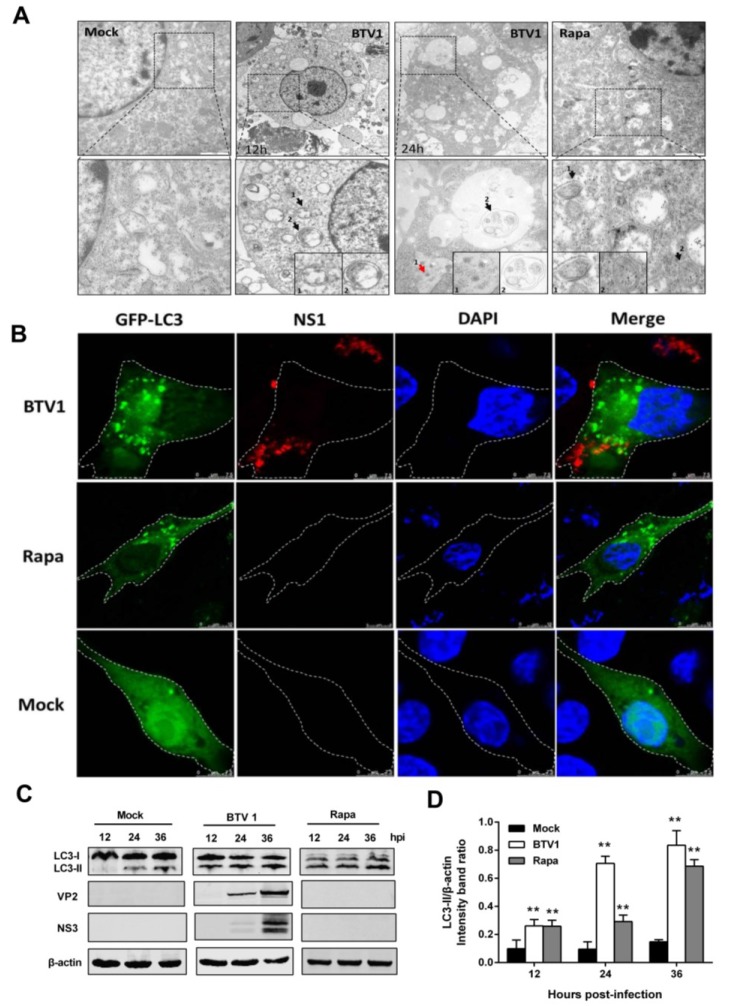
BTV1 infection triggers cellular autophagy in BSR cells. (**A**) TEM analysis. Mock-treated BSR cells as a negative control showed normal distribution of organelles. BSR cells infected with BTV1 at an MOI of 1 were harvested at 12 or 24 hpi and subjected to TEM observation. Cup-shaped membranous structures or autophagosome-like structures in the cytoplasm are indicated by the black arrows and BTV particles were pointed by red arrow. Higher-magnification views were listed below. Scale bars: 500 nm. Rapamycin-treated BSR cells were set as a positive control; (**B**) Confocal microscopy. Aggregation of autophagosomes is shown as green puncta in BSR cells that were transfected with GFP-LC3 followed by treatment with rapamycin (positive control); or infection of BTV1 at an MOI of 1 for 24 h at 24 h post-transfection. Mock treatment was a negative control. In addition, NS1 protein of BTV shown in red was used as indicator for BTV infection. The cell nucleuses were stained with DAPI. Scale bar: 7.5 μm. The outlines of the cells on the image were sketched by white dash line; (**C**) Western blotting. Transformation of LC3-II from LC3-I was detected in mock or BTV-infected BSR cells using an anti-LC3B antibody, and the expression of VP2 and NS3 of BTV1 was also detected using anti-VP2 and anti-NS3 antibodies, respectively. Rapamycin-treated group was positive control; (**D**) Intensity band ratios of LC3-II/β-actin. The values represent the mean ± SD of three independent experiments. Statistical significance was analyzed with Student’s *t*-test (* *p* < 0.05 and ** *p* < 0.01).

BTV1, the prevalent serotype of BTV in China, was selected to explore whether BTV can trigger autophagy in infected BSR cells. First, at 12 or 24 hpi, the formation of autophagosomes in BSR cells was examined by transmission electron microscopy (TEM), which is considered the most convincing approach for monitoring autophagosome induction [29]. As shown in Figure 1A, double-membrane vesicles were rarely observed in mock-infected cells. In contrast, in BTV1-infected cells, several double-membrane autophagic vesicles appeared, in which cytosolic components or organelles were sequestered. These double-membrane structures were similar to the autophagosomes in BSR cells induced by rapamycin, a well-known autophagy inducer, suggesting that the autophagosomes were induced in BSR cells by BTV1 infection.

Next, to further verify the observations above, the formation of GFP-LC3 dots was investigated after the pEGFP-LC3-transfected BSR cells were infected or mock-infected with BTV1 for 24 h. As presented in Figure 1B, NS1 expression of BTV1 firstly suggested the cells were indeed infected with BTV1, and a considerable number of punctate GFP-LC3 proteins accumulated in infected cells compared with the uninfected cells. The GFP-LC3 dots induced by rapamycin were used as a positive control.

The lipidated form of LC3 (LC3-II) is a hallmark of autophagy induction and is widely used to determine the presence of autophagy. In our study, the increase of LC3-II was monitored by immunoblotting at the indicated times after BTV infection. Rapamycin treatment was used as a positive control. As shown in Figure 1C, LC3-I-to-LC3-II conversion was significantly greater in BTV-infected cells relative to mock-infected cells. The ratio of LC3-II to β-actin as an indicator was significantly increased about seven times (*p* < 0.01) at 24 and 36 hpi (Figure 1D). Rapamycin-group was also displayed increased trend. Meanwhile, monoclonal antibodies that specifically recognize the BTV1, VP2 and NS3 proteins were used to track infection progression. Based on these experiments, we concluded that BTV1 infection triggered autophagy in BSR cells.

### 3.2. Autophagy is Induced in Primary Lamb Lingual Epithelial Cells by BTV1 Infection

**Figure 2:**
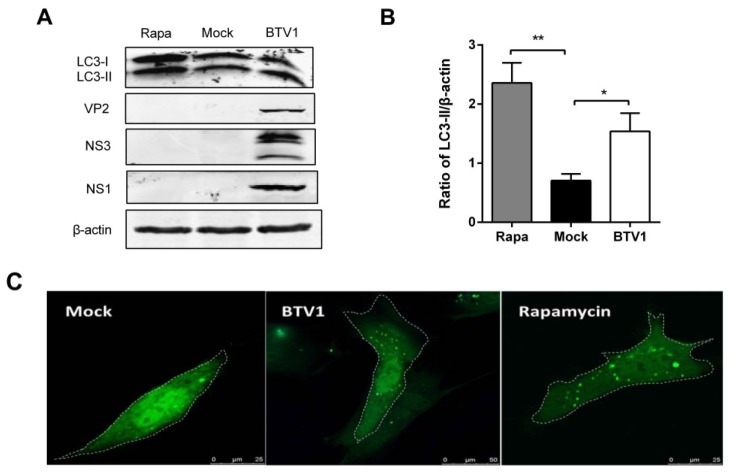
Autophagy was induced in primary lamb lingual epithelial cells by BTV1 infection. (**A**) The change from LC3-I to LC3-II in primary cells after BTV1 infection for 48 h was detected by Western blotting using an anti-LC3B antibody. Rapamycin group was positive control; (**B**) Ratios of LC3-II to β-actin of panel (**A**). The values represent the mean ± SD of three independent experiments. Statistical significance was analyzed with Student’s *t*-test (* *p* < 0.05); (**C**) The primary lamb lingual epithelial cells were transfected with pEGFP-LC3. At 24 h post-transfection, cells were treated with mock-infection (**left**) or BTV1-infection at an MOI of 1 for 48 h (**middle**); or rapamycin treatment for 36 h (**right**). The fluorescence signals were visualized by confocal microscopy. Scale bar: 7.5 μm.

Although BSR is a good model for the research of the pathogenesis of BTV, a cell line cannot completely replicate the physiology of host cells. Therefore, to explore whether autophagy is triggered by BTV1 infection in host cells, primary lamb lingual epithelial cells were used in this experiment. To firstly confirm BTV could infect and replicate in this cell, viral nonstructural proteins (NS1 and NS3) were detected at 48 h post-infection. In addition, the induced autophagy by BTV infection in the lamb lingual epithelial cells was assessed by both LC3-I-to-LC3-II conversion and GFP-LC3 dots as above. Rapamycin treatment was used as the positive control as above. Similarly, the results shown in Figure 2 indicated that both the LC3-II and the GFP-LC3 dots increased compared to the mock group after BTV1 infection, suggesting that autophagy is induced by BTV1 in lamb lingual epithelial cells.

### 3.3. BTV1 Infection Increases the Levels of Autophagic Flux

Autophagic flux is a continuous and complete process of autophagy. The fusion of lysosomes and autophagosomes and the degradation of substrates are essential components of autophagic flux, in addition to the formation of autophagosomes. p62, also known as Sequestosome-1 (SQSTM1), is an indicator to assess autophagic flux. When autophagic flux occurs, p62 can target specific cargoes for autophagy and is specifically degraded by the autophagic-lysosomal pathway [30].

**Figure 3:**
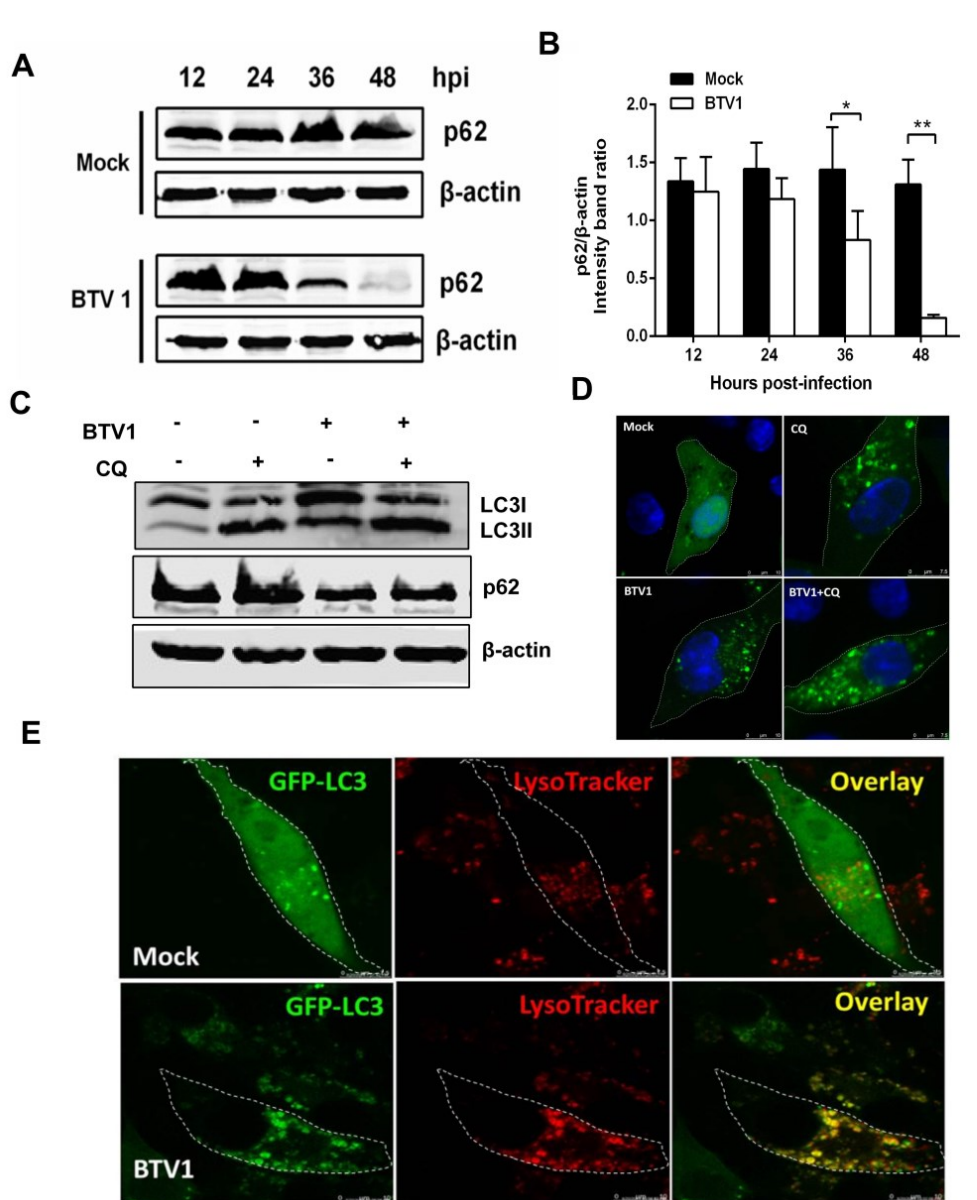
Autophagic flux measurement in BSR cells infected with BTV1. (**A**) p62/SQSTM1 (p62) degradation was tested by Western blotting. p62 in BTV1-infected and mock-infected BSR cells was monitored using an anti-p62 antibody; (**B**) Representative graphs were shown exhibiting the ratios of p62 to β-actin (mean ± SD) from three independent experiments. Statistical significance was analyzed with Student’s t test (* *p* < 0.05 and ** *p* < 0.01); (**C**) The expression levels of LC3-II and p62 were detected by Western blotting after mock-infection or BTV1-infection in the presence or absence of 20 μM CQ for 24 h; (**D**) Representative confocal images of BTV with or without CQ treatment for 24 h; (**E**) Representative confocal images of co-localization of the autophagosome marker GFP-LC3 (**green**) with the acidic lysosome marker LysoTracker (**red**) in BSR cells infected with BTV1 for 36 h. Mock-infected cells served as negative control. Scale bar: 7.5 μm.

To investigate whether BTV infection induces autophagic flux, we measured p62 degradation levels. Immunoblotting revealed significant progressive degradation of p62 in BTV-infected cells compared with the unaltered p62 levels in mock-infected cells (Figure 3A), suggesting that autophagosomes were able to fuse with lysosomes to degrade the cargos. The ratios of p62 to β-actin shown in Figure 3B indicated that p62 reduction was notable at 36 hpi (about one time) and peaked at 48 hpi (about five times).

Additionally, the turnover of LC3-II caused by the inhibition of autophagic degradation further indicated autophagic flux. For this reason, we detected the expression of LC3-II and p62 with or without CQ, an inhibitor of lysosomal acidification and autophagic proteolysis, by Western blotting. As shown in Figure 3C, the accumulation of LC3-II and p62/SQSTM1 was observed with the employment of CQ at 24 h post-infection in BTV-infected cells compared with untreated controls, further indicating enhanced autophagic flux. The confocal image of BTV plus CQ also showed more undegraded and accumulated LC3 puncta in Figure 3D.

Lastly, the fusion of autophagosomes with lysosomes was verified by labeling lysosomes with an acidified compartments marker LysoTracker (red fluorescence) [31,32]. As demonstrated in Figure 3E, the colocalization of GFP-LC3-tagged autophagosomes and LysoTracker-stained lysosomes was detected in BTV1-infected BSR cells, while uninfected cells exhibited almost no overlap. These results indicated that cells underwent a complete autophagic process following BTV infection.

### 3.4. The Autophagy is Dependent on BTV-Productive Infection

**Figure 4:**
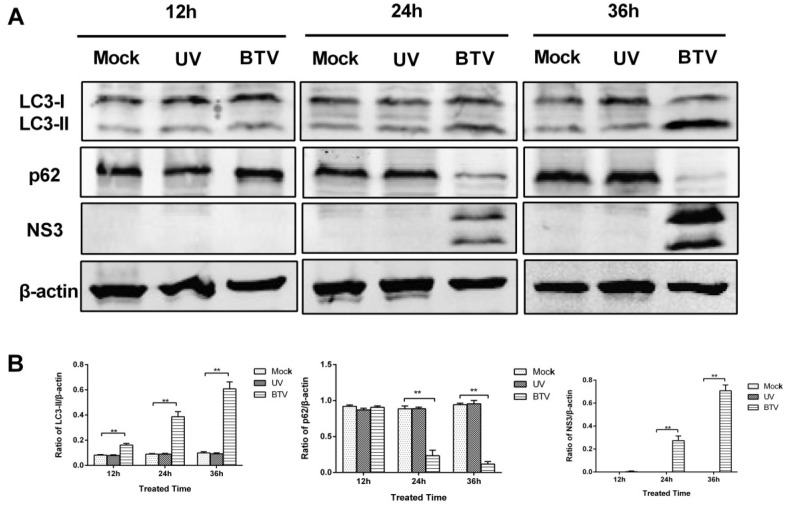
The induction of autophagy by BTV1 was dependent on productive infection. (**A**) BSR cells were untreated (**left**) or incubated with UV-irradiated BTV1 (**middle**) or normal BTV1 (**right**) for the indicated amounts of time. Afterwards, to determine the induction of autophagy, the cells were lysed to detect LC3, p62, β-actin and NS3 of BTV using corresponding antibodies; (**B**) Representative results are shown with graphs representing the intensity of LC3-II/actin or p62/actin or NS3/actin bands. Data represent mean ± SD of three independent experiments (** *p* < 0.01 and * *p* < 0.05).

Based on the results of LC3 lipidation and p62 degradation, we found that autophagy became more and more apparent with BTV replication, and we wondered if the induction of autophagy by BTV required productive infection. First, infective BTV1 was inactivated by UV irradiation. Then, BSR cells were infected with this UV-irradiated BTV1 for the indicated amounts of time, and LC3, p62 and NS3 in the cells were detected by Western blot. Compared with the normal BTV1 infection, neither virus-induced detectable LC3-I-to-LC3-II conversion nor the expression of viral NS3 protein was detected at any time point post-infection of UV-irradiated BTV1. Additionally, p62 degradation did not appear with UV-irradiated BTV1 infection (Figure 4). Taken together, these results suggested that the autophagy induced by BTV was dependent on viral replication.

### 3.5. Inhibition of Autophagy Reduced BTV1 Replication

**Figure 5:**
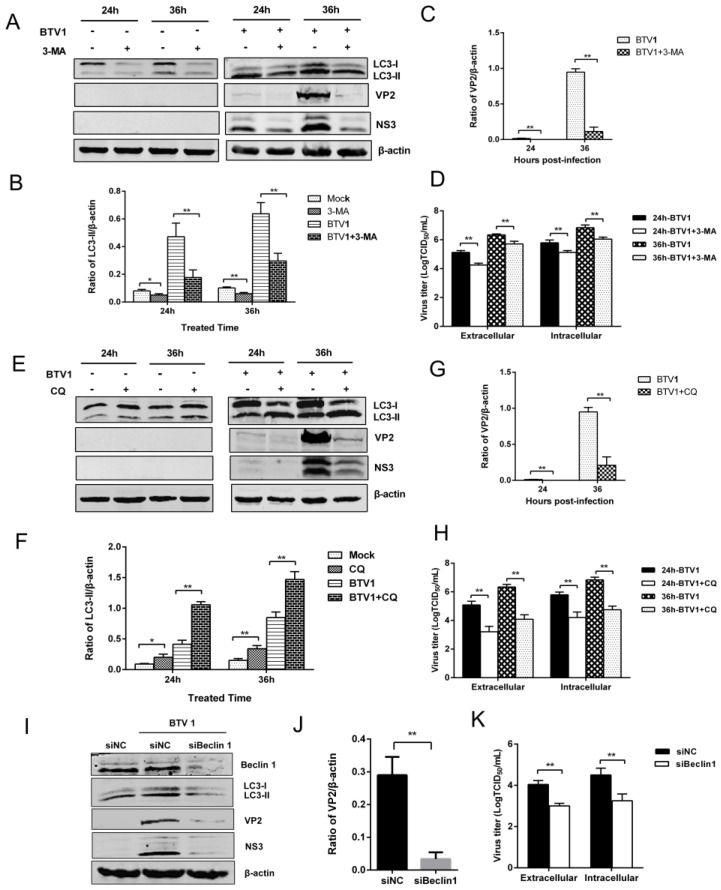
Autophagy inhibition reduced the replication of BTV1. (**A**,**E**) Effects of 10 mM 3-MA or 20 μM CQ treatment on LC3-II accumulation and VP2 or NS3 expression. The expression of relative proteins were analyzed by immunoblotting with specific antibodies; (**B**,**F**) Representative results are shown with graphs representing the intensity of LC3-II/actin; (**C**,**G**) Representative results are shown with graphs representing the intensity of VP2/actin. (**D**,**H**) Effects of 10 mM 3-MA or 20 μM CQ on the production of infectious particles of BTV1. The extracellular and intracellular virus loads were tested as TCID_50_/mL at 24 hpi and 36 hpi, respectively; (**I**) The effects of Beclin1 silencing on LC3-II and VP2 or NS3 expression. BSR cells were transfected with either specific siRNA targeting Beclin1 or negative control siRNA. At 24 h after transfection, the cells were incubated with BTV1 (MOI = 1). Samples were collected 24 hpi and analyzed by Western blotting with antibodies against Beclin1, LC3, VP2, and NS3; (**J**) Representative results are shown with graphs representing the intensity of VP2/actin; (**K**) The viral titers were determined as TCID_50_/mL at 24 hpi. Data represent the mean ± SD of three independent experiments. * *p* < 0.05 and ** *p* < 0.01, significantly different.

Given that the induction of autophagy was closely related to BTV1 replication, we asked the question: What role does autophagy play in the BTV1 life cycle? To answer this question, the effects of autophagy on the replication of BTV were analyzed by inhibiting autophagy. 3-MA is a widely used class III phostphatidylinositol-3-kinase (PI3K) inhibitor that disturbs early autophagosome formation [33]. Thus, 3-MA was first used to investigate the replication of BTV after early autophagosome disturbance. As the results show (Figure 5A,B), the induced-LC3-II was inhibited by 3-MA treatment at the optimal concentration (10 mM) with no cytotoxicity (Figure 7; Lane 2). Meanwhile, expression of the viral proteins VP2 was reduced about seven times in autophagy-inhibited BSR cells compared to control cells (Figure 5C). Similarly, viruses of supernatants or freeze-thaw cells were collected from 3-MA-treated cells at the indicated times. The extracellular virus titers were decreased nearly 1.28 folds at 24 hpi and 1.14 folds at 36 hpi, and the intracellular virus titers were also reduced similarly, (Figure 5D), suggesting that early autophagy was beneficial to BTV growth.

Late autophagy, characterized by autophagosome-lysosome fusion, is also an essential step for the autophagic process. To investigate the impact of late autophagy in virus replication, BSR cells were treated with CQ at an optimal concentration (20 μM) to inhibit autophagy through blocking autophagosome-lysosome fusion. As demonstrated in Figure 5E, F, compared with the control group, there was significant accumulation of LC3-II in CQ-treated cells with no cytotoxicity (Figure 7; Lane 3). Moreover, this figure shows a six-fold decreased expression of VP2 protein in the cells at 36 hpi (Figure 5G). Similarly, extracellular and intracellular virus yields at 24 hpi and 36 hpi decreased nearly 2 folds with CQ treatment (Figure 5H), indicating that autophagy inhibition at late stage also dramatically reduced BTV replication.

To exclude any non-specific effects of these pharmacological experiments, RNA interference was used to suppress the expression of Beclin1, which is at the heart of a regulatory complex during autophagosome formation and maturation. As shown in Figure 5I, compared with negative control siRNA-transfected cells, BSR cells transfected with Beclin1-targeting siRNAs exhibited a notable decrease in endogenous Beclin1 protein with no cytotoxicity (Figure 7; Lane 6). LC3-II formation was also greatly reduced by the suppression of Beclin1 expression. Furthermore, Beclin1 silencing was accompanied by significantly reduced viral protein synthesis, as the ratio of VP2/actin presents nearly nine-fold decrease and extracellular and intracellular virus particle yields also showed more than 1.5-fold reduction at 24 hpi (Figure 5J, K).

These results were consistent with previous pharmacological studies. Taken together, the drug inhibition and Beclin1 interference suggest that autophagy was involved in and required for the effective replication of BTV.

### 3.6. Induction of Autophagy Promotes BTV1 Replication

**Figure 6:**
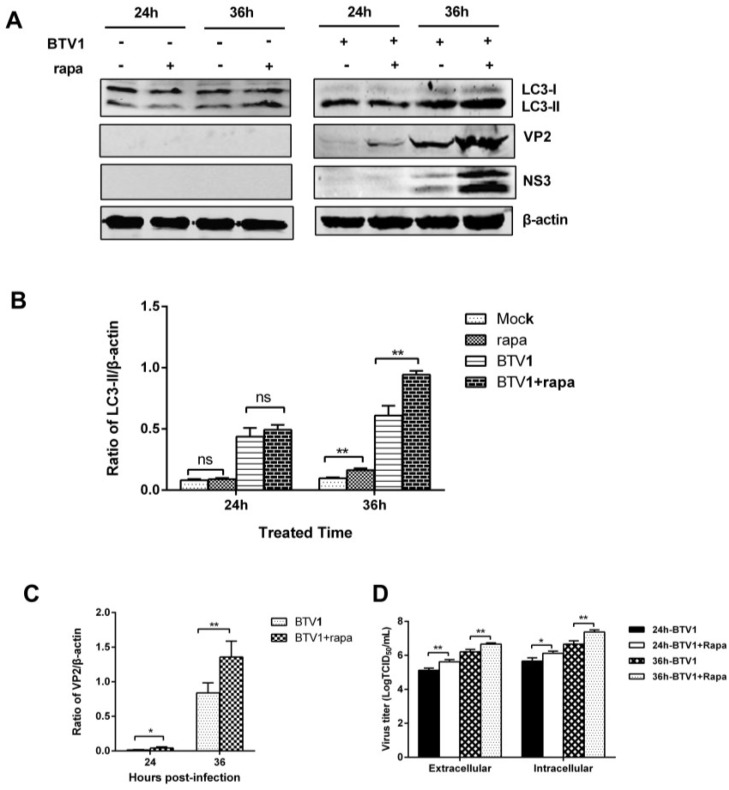
Induction of autophagy with rapamycin enhanced the replication of BTV1. (**A**) Effects of 100 nM rapamycin treatment on LC3-II accumulation and VP2 or NS3 expression. The intensity ratios of proteins relative to β-actin are presented below the blots; (**B**,**C**) Representative results are shown with graphs representing the intensity of LC3-II/actin or VP2/actin bands; (**D**) The titers of BTV1 produced by rapamycin-treated BSR cells. The virus yields were shown as TCID_50_/mL at 24 hpi and 36 hpi. Results represent the mean ± SD of triplicate experiments. * *p* < 0.05 and ** *p* < 0.01, significantly different; ns, no significant difference.

**Figure 7:**
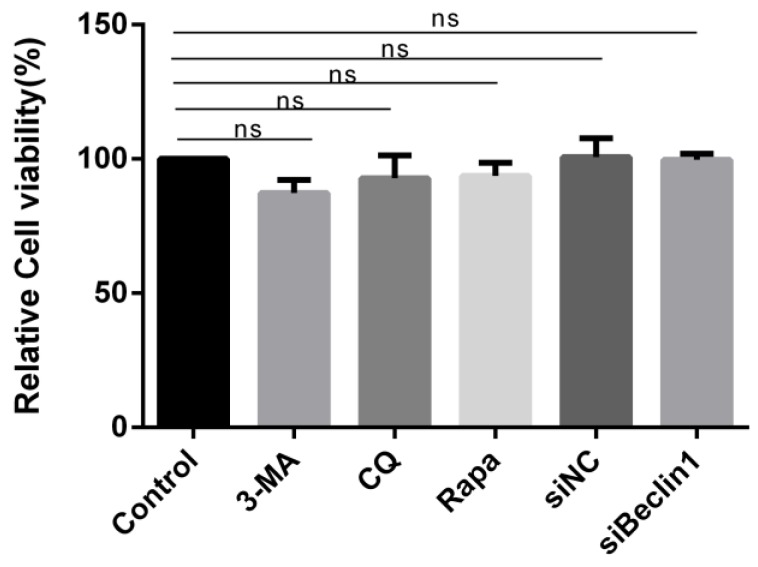
Pharmacological or siRNA treatments had no impact on cell viability. Cell viability was detected by WST-1 assay after treatments with 3-methyladenine (3-MA), chloroquine (CQ), rapamycin (Rapa), or transfection with siBeclin1 for 48 h. Light absorption at 450 nm was recorded and expressed as percentage of relative cell viability, the values represented mean ± SD. “ns” means no significant difference, *p* > 0.05.

Our suppressive results prompted further exploration of the relationship between activated autophagy and virus replication. Thus, we probed for the effects of autophagy induction on viral yield and viral protein expression. Rapamycin, an autophagy inducer that directly inhibits the action of mTOR [34], was used as a positive indictor in autophagy study. LC3-II presented a rising trend in rapamycin-treated cells (Figure 6A) with no cytotoxicity (Figure 7; Lane 4) compared to the control. Viral protein synthesis levels were promoted by enhanced autophagy induction, as reflected in the increased expression of VP2 and NS3 compared with the control group, especially at 36 hpi (Figure 6A,C). Furthermore, enhanced autophagy also markedly increased the yield of BTV progeny at 24 hpi and 36 hpi in extracellular and intracellular virus samples, showing about 1-fold increment (Figure 6D). In brief, these data indicate that enhanced autophagy is favorable for the replication of BTV in BSR cells.

### 3.7. Cell Viability Unaffected by Pharmacological Treatment

Considering that high concentrations of drugs or knockdown of Beclin1 might influence cell viability and thus affect our results, the effects of the compounds used in this study on cell viability were analyzed in a WST-1 assay. The data show that the viability of BSR cells was hardly affected by siRNA transfection or drug treatment at the indicated effective doses (Figure 7). These experiments demonstrate the effectiveness of pharmacological treatment and RNA interference on studies of autophagy and BTV replication.

## 4. Discussion

BTV causes a severe disease in ruminant animals and has a major influence on livestock economy. Previously, our understanding of BTV mostly focused on molecular structure, global epidemiology and diagnostic methods; however, the interaction between BTV infection and the host cell response has not been well studied. Recently, autophagy as a cellular adaptive response has been found to be involved in various viral infections [35,36]. However, the relationship between BTV infection and autophagy has not been thoroughly examined.

In this study, we observed that BTV1 infection triggers an increase in autophagosome-like double-membrane vesicles, the accumulation of GFP-LC3 puncta, and the conversion of LC3-I to LC3-II in permissive BSR cells (Figure 1), indicating that autophagosomes were formed [37]. Moreover, we identified similar autophagic phenomena during BTV1 infection in natural target cells: primary lamb lingual epithelial cells (Figure 2). These observations of natural target cells provide a valuable reference for the study of the interactions between BTV infection and the host cell response. Overall, we concluded that BTV infection triggered autophagosomes formation in host cells.

Autophagic flux is dynamic and continuous, refers to the increased autophagosome formation as well as the entire process, including lysosomes fusion and substrate recycle, thus, autophagic flux is a more accurate indicator of autophagy activity than autophagosomes formation [37]. Notably, the autophagic response to different viruses is not always the same. The autophagic flux induced by some viruses is incomplete, such as with porcine reproductive and respiratory syndrome virus and hepatitis C virus (HCV) [38,39], which implies that autophagic flux is virus specific. Our results show that BTV1 infection promoted p62 degradation (Figure 3A), and the lysosome inhibitor CQ increased the levels of LC3-II and p62 in virus-infected cells (Figure 3C). Furthermore, colocalization of GFP-LC3 with LysoTracker in infected cells was observed (Figure 3E). These findings demonstrated that complete autophagic flux was triggered upon BTV infection.

The finding that LC3-II formation and p62 degradation were enhanced by BTV replication implies the autophagy induced by BTV was strictly dependent on productive infection. Indeed, the same phenomena were not observed when BSR cells were incubated with the UV-irradiated BTV1 virus (Figure 4) that lacks replication ability. UV-inactivated BTV could not induce autophagy.

Given that viral replication was required for BTV infection-induced autophagy, what is the effect of autophagy on BTV replication? Autophagy is a balancing mechanism that maintains the homeostasis of cells. Although autophagy commonly serves as a defense mechanism, growing evidence suggests that some RNA viruses utilize or manipulate this cellular process to facilitate their own survival and replication [17,40], for instance influenza A virus [18], epizootic hemorrhagic disease virus [20], and avian reovirus [22]. To define the role of autophagy in BTV1 replication and provide a basis for further study of the relationship between autophagy and BTV, we analyzed the physiological significance of autophagy on BTV1 replication *in vitro*. Pharmacological treatments, including early autophagic inhibitor 3-MA and late autophagic inhibitor CQ, significantly downregulated viral protein expression and virus production. Furthermore, siRNA-mediated silencing of Beclin1 resulted in effects on BTV1 replication similar to those induced by 3-MA and CQ (Figure 5). Correspondingly, the expression of viral proteins and the production of infectious virions were promoted by exposing infected cells to the autophagy inducer rapamycin (Figure 6). Therefore, these results definitively demonstrated that autophagy is a critical process activated by bluetongue virus that benefits its replication.

It is worth mentioning that CQ used in the above assays is a multifunctional drug in biological tests, whose functions vary by dosage and usage. According to reference [41], CQ was also used to block clathrin-mediated endocytosis at a dosage of 200 μM, although we applied CQ as inhibitor of lysosomal acidification and autophagic proteolysis at a concentration of 20 μM during the whole process of virus infection, it is hard to know whether 20 μM CQ pretreatment has effect on endocytosis, which is the entry method used by BTV infection. Therefore, to rule out the possibility that 20 μM CQ pretreatment will affect BTV entry and accurately assess the effect of autophagy on BTV replication, BSR cells in our assays were not pretreated with 20 μM CQ.

Many viruses have evolved to exploit specific components of autophagy to assist in viral replication. Firstly, the autophagosome as a typical membrane vesicle structure could serve as the virus factory or viroplasm for many viruses. The virus factories or viroplasms are special structures that provide a physical scaffold to concentrate viral components and thereby facilitate virus replication and assembly [42]. For example, a study recently found that classical swine fever virus (CSFV) localized at the autophagosome-like vesicle membrane, which was involved in the replication of CSFV [39]. Additionally, a rotavirus pore-forming protein activates a calcium-dependent signaling pathway to initiate autophagy and hijacks this membrane trafficking pathway to transport viral proteins to viroplasms, the site of genome replication and viral assembly [43]. Viroplasms are characteristic of reovirus infections in general [44]. Given that BTV is a member of *Reoviridae* and its encoded proteins serve a similar function to rotavirus, we speculated that the vesicle membrane might also be involved in the replication of BTV. Thus, more work is underway to study how BTV takes advantage of host autophagy (or components of autophagy).

Second, vesicular acidification, which precedes the delivery of cargo to lysosomes during autophagy, may provide an environment that is beneficial to virus production. Autolysosomes formed by the fusion of autophagosomes with lysosomes are the main site for autophagic degradation. The acidic environment in autolysosomes is essential for the degradation or modification of contents trapped in the autophagosome. For some viruses, these events related to vesicular acidification are used to assist in their replication. For example, the production of the infectious poliovirus virion depends on vesicle acidification to modify the capsid protein VP0 [45]. In addition, degraded host components could also be used by the virus. For example, lipid droplets from the degradation of cholesterol in autolysosomes are required for HCV assembly [46]. For BTV, the release of the BTV core is dependent on an acidic pH [47], and accumulating evidence suggests that VP5 is involved in the permeabilization of the endosomal membrane in a pH-dependent manner [48]. An inhibitor of endosomal acidification, CQ, significantly reduced the replication of the virus. Thus, BTV could utilize the acidic environment during late autophagy for its own benefit.

In conclusion, our findings suggest that autophagy is triggered upon BTV infection and is critical for viral replication, implying that the autophagic pathway may be utilized to sustain BTV replication. Clearly, our understanding of the molecular mechanisms driving the interplay between BTV and autophagy is still insufficient, as several aspects of BTV-induced autophagy remain unclear, but the basic data presented here are the first step in our exploration. Our studies provide novel insights into BTV-host interactions and open a new window to examine the pathogenesis of BTV. This knowledge will contribute to the development of antiviral strategies or drugs against BTV infection.
