# Correction: Shuang, L.; *et al*. Autophagy Activated by Bluetongue Virus Infection Plays a Positive Role in Its Replication, *Viruses* 2015, 7, 4657–4675

**DOI:** 10.3390/v8040089

**Published:** 2016-03-24

**Authors:** Viruses Editorial Office

**Affiliations:** MDPI AG, Klybeckstrasse 64, CH-4057 Basel, Switzerland

The *Viruses* Editorial Office wishes to notify its readers of a correction in [1]. The third paragraph of Section 2.5 should be replaced with the following:

“To detect autophagic flux, BSR cells were transfected with pEGFP-LC3, infected with BTV or left untreated as described above for the indicated time. Then the cells were stained with 50 nM LysoTracker Red DND-99 (acidic compartments marker) (C1046, Beyotime) for 30 min. After the cells were washed three times with PBS, they were processed for analysis using a confocal microscope”.

The manuscript will be updated and the original will remain available on the article webpage. The authors would like to apologize for any inconvenience caused.
